# Supplementary material for: Amyloid-like Hfq interaction with single-stranded DNA: involvement in recombination and replication in Escherichia coli
Source: QRB Discov. 2022 Sep 7;3:e15. doi: 10.1017/qrd.2022.15 (PMC10392684; doi:10.1017/qrd.2022.15)
Supplement: Supplementary file 1 [file qrdsup.zip › S2633289222000151sup001.docx]

**METHODS**

***Protein expression and purification***

Wild type (WT) and truncated Hfq (residues 1 to 65, referred to as NTR through the manuscript) forms of *Escherichia coli* Hfq were purified as described previously(Malabirade et al., 2017; Taghbalout et al., 2014). Hfq C-terminal (CTR) peptide corresponding to the amyloid domain of Hfq (residues 64 to 102, referred as CTR) was synthetized by Proteogenix SA (France). This part of the protein cannot be purified from bacteria as it is unstable when translated alone in bacteria(Taghbalout et al., 2014). The sequence of the CTR peptide was SRPVSHHSNNAGGGTSSNYHHGSSAQNTSAQQDSEETE(Fortas et al., 2015). CTR peptides were reconstituted in water at 20 mg/mL. We determined that the pH used in our condition (~ 5) was the most appropriate to form the complex with DNA. Indeed, the positive charge of the peptide at pH 5 allows its interaction with nucleic acids, while increasing pH abolishes this interaction. We also chose to avoid the addition of salts (except those already present in DNA and peptide solutions) in order to allow a better investigation in deep-UV(Malabirade et al., 2018)when the complex is analysed in the presence of salts (or far-UV absorbing buffers), the spectral bandwidth accessible is limited, reducing the spectral information obtained(Le Brun et al., 2020; Wien et al., 2019).

***Fluorescence anisotropy measurements***

Fluorescence anisotropy measurements were collected with a Varian Eclipse fluorospectrophotometer, as described previously(Geinguenaud et al., 2011). Briefly, samples were excited at 490 nm and emission was measured at 520 nm. 5’-fluoresceinated dA_7_, dA_20_ and dA_59_ oligonucleotides were purchased from Eurogentec (Belgium). 1 nM of 5’-fluoresceinated oligonucleotide was added to the cuvette (1 ml) and titrated by Hfq or its truncated forms. Samples were incubated 60 s prior to each measurement, ensuring equilibrium binding. The normalization of fluorescence anisotropy was carried out after determination of the Amax value, which was obtained at saturating Hfq concentrations. A/Amax ratios were plotted versus the Hfq concentrations. The curves were fitted by the non-linear least-squares regression method, assuming a bimolecular model with Hfq hexamer as protein unit for full length and NTR Hfq (the biological form of the protein), and monomer unit for the CTR. Binding affinities were measured for 3 different experiments. Note that binding of Hfq to dA_59_ (unlabelled and labelled) was also confirmed by EMSA to ensure the presence of the fluorescence label does not influence ssDNA binding.

***Optical Microscopy of ssDNA-Hfq/CTR/NTR Complexes***

Sample preparation: DNA in the single-stranded form was prepared by alkali-induced denaturation of double-stranded λ-DNA(Basak et al., 2019). For this, 180 μL of 0.5 M NaOH was added to 15 μL of 80 mg of λ-DNA/L in TE- buffer (Tris-HCl 10 mM pH8, EDTA 1 mM) and incubated overnight at 277 K. After that, the nucleoid associated protein Hfq, 2 mg/mL (29.8 µM) (dissolved in Tris-HCl 50 mM pH 7.5, NH_4_Cl 50 mM, glycerol 10%, EDTA 1 mM) was added to the solution with concentration of one hexamer-Hfq per 200 bases of DNA. Subsequently, the alkali buffer was replaced by TE buffer through six-times repeated centrifugation (12,000 g, 20 min per run, 278 K) and by using a 3 kDa cut-off membrane. After each run, the precipitate was topped up with 400 μL of TE-buffer. A similar procedure was used to make the ssDNA with Hfq-CTR and Hfq-NTR. However, the molar concentration of CTR used was six times higher than Hfq, i.e. 6 CTR for 200 bases of DNA. The final ssDNA concentration was 3 mg/L.

Fluorescence imaging: About one hour before imaging, ssDNA was stained with YOYO-1 at a concentration of one YOYO-1 dye per four bases. For molecular combing, polystyrene (MW = 280 kDa, Sigma Aldrich) was dissolved in toluene (Fisher Scientific, Pittsburgh, PA) at a concentration of 1 g/mL. Cleaned glass cover slips were spin-coated with the polystyrene solution for 30 s at 2000 rpm. A 2 µL droplet was spotted on the cover slip and sheared in a linear manner along the surface with a pipette tip. For nanofluidic measurements, devices featuring rectangular-shaped channels with a length of 90 μm, a depth of 130±5 nm, and a width of 120±5 nm were made of polydimethylsiloxane with enhanced elasticity modulus (X-PDMS)(Yadav et al., 2020). The average cross section diameter of the channels is 125 microns. Video recording was started 2 min after the molecules were brought into the channels using electrophoresis. The exposure time of the camera for both cases (combing and nanofluidic) was 300 ms and no anti-photo bleaching agent was used for either case.

***Preparation of the complexes for Circular Dichroism (SRCD) and Infrared (FTIR) spectroscopies***

Complexes between ssDNA dA_59_ (Eurogentec) and Hfq-CTR peptides were prepared as described previously for dsDNA(El Hamoui et al., 2020; Malabirade et al., 2018; Turbant et al., 2021). Briefly, complexes between Hfq-CTR and ssDNA were prepared and used at a final concentration of 1.8 mM and 7.3 mM, respectively. The stoichiometry was 1 Hfq-CTR per 4 base pair. Samples were analyzed up to 2 weeks after preparation to allow peptide self-assembly on DNA that is not instantaneous(Malabirade et al., 2018).

***Synchrotron Radiation Circular and Linear Dichroism (SRCD and SRLD)***

SRCD and SRLD measurements were carried out on DISCO beamline at SOLEIL Synchrotron (proposal 20200007) previously described(Malabirade et al., 2018; Refregiers et al., 2012). Samples (~ 4 µl) were loaded into a CaF_2_ circular cell of 24 µm pathlength(Wien & Wallace, 2005). Spectral acquisitions of 1 nm steps at 1.2 s integration time were recorded in triplicate between 320 and 170 nm. (+)-camphor-10-sulfonic acid (CSA) was used to calibrate amplitudes and wavelength positions of the experiment. Data analyses (averaging, baseline subtraction, smoothing, scaling and spectral summations) were carried out with CDtool(Lees et al., 2004). Spectra are presented in units of mdeg versus nm maintaining the same molar ratios for all presented samples. Due to the origin of absorption, spectra of mixed samples (polynucleotide + peptides) could not be standardized to ∆ε.

SRLD measurements were carried out in the same cell of 24 µm pathlength using the automated rotation chamber(Wien et al., 2013), collecting triplicates every 90° from 0-270°. For the data-acquisition the modulator phase was set to λ x 0.608 doubling the lock-in amplifier frequency, in order to measure only LD absorption.

***Couette flow Synchrotron Radiation Linear Dichroism (SRLD)***

Couette flow SRLD measurements were performed at the AU-CD beamline on the ASTRID2 synchrotron (proposal ISA-21-102). The samples were aligned for LD measurements under Couette flow conditions, where a 3.0/2.5 mm outer/inner diameter Suprasil capillary was rotated around a 2.0 mm outer diameter stationary Suprasil rod, leaving a path length gap of 0.5 mm for the sample(Marrington et al., 2005; Wien et al., 2019). The Couette flow cell was loaded with 60 µL sample, a baseline spectrum was acquired without rotation followed by the LD measurement recorded with a rotation speed of 3000 rpm. The spectra were recorded between 180 and 350 nm in 1 nm steps and a 2 sec dwell time per point. The final LD spectrum is the spectrum recorded with rotation with the non-rotation spectrum subtracted. Due to the much larger path length of the Couette flow cell used for SRLD measurements (0.5 mm) compared to the path length used for SRCD measurements (0.024 mm), the complex between Hfq-CTR and the ssDNA (dA_59_) had a very strong LD signal under flow conditions; the samples where diluted (1/36) compared to the concentrations used for SRCD.

***Fourier Transform Infrared spectroscopy (FTIR)***

For FTIR analysis, the same solutions used for SRCD analysis were lyophilized and re-dissolved in D_2_O (5 µL). Note that the low pH used (see above) could result in partial protonation of our ssDNA A strand, but this does not impair complex formation. FTIR spectra were recorded using a Bruker Tensor 27 spectrophotometer. For transmission experiments, samples were deposited between two ZnSe windows without spacer. 30 scans were usually accumulated under continuous dry air purging, with a resolution of 1 cm^-1^. Data treatment was performed using the OPUS software (Bruker).

***TEM imaging***

ΦX174 ssDNA (5386 nt) was diluted to a concentration of 6.5 µg/ml in TE buffer (10 mM Tris-HCl containing 1 mM EDTA) with a CTR concentration of 100 nM and incubation was carried out for 10 minutes at room temperature. 5 μl of the reaction solution were deposited onto a 600-mesh copper grid covered with a thin carbon film and activated by glow-discharge in the presence of pentylamine (Beloin et al., 2003). Grids were washed with aqueous 2% (w/v) uranyl acetate, dried and observed in annular darkfield mode using a Zeiss 902 electron microscope. Images were captured with a Veletta CCD camera controlled by iTEM software (Olympus Soft Imaging).

***E. coli strains and bacteriophages***

*E. coli* wild-type strain MG1655(Jensen, 1993) was used as the *hfq*^+^ variant (control). Its Δ*hfq* and ΔCTR derivatives were constructed using the λ Red system as described in Gaffke *et al*(Gaffke et al., 2021). Quantification of Hfq (full length) and its CTR-truncated form was performed using Western-blotting(Gaffke et al., 2021) and confirmed by dot-blotting for better statistics.

For propagation of bacteriophage M13, *hfq*^+^, Δ*hfq* and ΔCTR derivatives of *E. coli* strain Hfr3000 (Bachmann, 1972) were constructed by P1 transduction. Bacteriophages M13(Salivar et al., 1964), λ*c*I857*S*7(am)(Goldberg & Howe, 1969), called λ*S*(am) in this work, and λ*b519imm21susP*(Wegrzyn et al., 1995)*,* called λ*P*(am) in this work, were used. *E. coli* strain TAP90 (F^-^ *recD1903::mini-tet supE44 supF58 lacY1 pro leuB6 hsdR rpsL tonA1 thi-1*)(Patterson & Dean, 1987) was used for propagation and titration of phages λ*cI857S7*(am) and λ*b519imm21susP.*

***Bacteriophage M13 development***

Overnight bacterial culture growing in LB media was diluted 1:100 in a fresh medium, and the culture was grown with shaking at 37^o^C to A_600_ of 0.2. Following 5 min incubation without agitation, the culture was chilled to 4^o^C for 10 min. M13 phage lysate was added to m.o.i. = 1. After 8 min incubation (during this time a few 0.1 ml samples were withdrawn, centrifuged for 1 min at 12,000 *g*, and supernatants were stored at 4^o^C before titration), the culture was centrifuged for 3 min at 7,500 *g*, and the pellet was suspended in 2 ml LB. Addition of 18 ml LB, prewarmed to 37^o^C, was assessed as time 0. Cultivation was continued with shaking at 37^o^C for 60 min. Samples (0.1 ml each) were withdrawn at indicated times, centrifuged for 1 min at 12,000 *g*, and supernatants were stored at 4^o^C before titration. Phage titration of all samples was performed using the Hfr3000 strain as indicator. Phage titers were calculated considering number of plaques on plates after overnight incubation at 37^o^C. The results are presented as number of plaque forming units (pfu) per one infected cell (determined by mixing of 0.1 ml of serial dilutions of samples withdrawn at time=0 with 0.1 ml of the culture of the indicator strain and 3 ml of nutrient broth containing 0.7% agar, pouring on a Petri dish with LB containing 1.5% agar, and incubating overnight at 37 ^o^C followed by counting of plates).

***Efficiency of phage λ recombination***

Overnight bacterial culture growing in LB was diluted 1:100 in a fresh medium containing 10 mM CaCl_2_ and 10 mM MgSO_4_, and the culture was grown with shaking at 37^o^C to A_600_ of 0.2. The culture was centrifuged at 5,000 *g* for 10 min, and the pellet was suspended in 2 ml of TM buffer (10 mM Tris-HCl, 10 mM MgSO_4_, pH 7.2). Lysates of bacteriophages λ*cI857S7*(am) and λ*b519imm21susP* were added, either together or separately, to m.o.i. of 5 each. Following 15 min incubation in the ice bath, the mixtures were transferred to 37^o^C for 3 min, centrifuged at 5,000 *g* for 10 min at 4^o^C, and washed twice with TM buffer. After final washing, the pellet was suspended in 10 ml of LB prewarmed to 37^o^C, and cultivation was conducted at this temperature, with shaking, for 60 min. Then, 2 ml of chloroform was added, and the sample was shaken in vortex for 1 min. Following centrifugation at 8,000 *g* for 10 min, supernatant (phage lysate) was titrated using *E. coli* MG1655 (wild-type) and TAP90 (*supE supF*) strains. Phage titers were calculated considering number of plaques, and fractions of recombinants were calculated. Relative efficiency of recombination is shown assuming the value obtained for the wild-type host to be 100%.

***References***

Bachmann, B. J. (1972). Pedigrees Of Some Mutant Strains Of Escherichia Coli K-12. Bacteriological Reviews, 36(4), 525-557.

Basak, R., Liu, F., Qureshi, S., Gupta, N., Zhang, C., De Vries, R., Van Kan, J. A., Dheen, S. T. & Van Der Maarel, J. R. C. (2019). Linearization And Labeling Of Single-Stranded DNA For Optical Sequence Analysis. J Phys Chem Lett, 10(3), 316-321.

Beloin, C., Jeusset, J., Revet, B., Mirambeau, G., Le Hegarat, F. & Le Cam, E. (2003). Contribution Of DNA Conformation And Topology In Right-Handed DNA Wrapping By The Bacillus Subtilis Lrpc Protein. J Biol Chem, 278(7), 5333-5342.

El Hamoui, O., Yadav, I., Radiom, M., Wien, F., Berret, J.-F., Van Der Maarel, J. R. C. & Arluison, V. (2020). Interactions Between DNA And The Hfq Amyloid-Like Region Trigger A Viscoelastic Response. Biomacromolecules, 21(9), 3668-3677.

Fortas, E., Piccirilli, F., Malabirade, A., Militello, V., Trepout, S., Marco, S., Taghbalout, A. & Arluison, V. (2015). New Insight Into The Structure And Function Of Hfq C-Terminus. Biosci Rep, 35(2).

Gaffke, L., Kubiak, K., Cyske, Z. & Wegrzyn, G. (2021). Differential Chromosome- And Plasmid-Borne Resistance Of Escherichia Coli Hfq Mutants To High Concentrations Of Various Antibiotics. Int J Mol Sci, 22(16), 8886.

Geinguenaud, F., Calandrini, V., Teixeira, J., Mayer, C., Liquier, J., Lavelle, C. & Arluison, V. (2011). Conformational Transition Of DNA Bound To Hfq Probed By Infrared Spectroscopy. Phys Chem Chem Phys, 13(3), 1222-1229.

Goldberg, A. R. & Howe, M. (1969). New Mutations In The S Cistron Of Bacteriophage Lambda Affecting Host Cell Lysis. Virology, 38(1), 200-202.

Jensen, K. F. (1993). The Escherichia Coli K-12 "Wild Types" W3110 And MG1655 Have An Rph Frameshift Mutation That Leads To Pyrimidine Starvation Due To Low Pyre Expression Levels. J Bacteriol, 175(11), 3401-3407.

Le Brun, E., Arluison, V. & Wien, F. (2020). Application Of Synchrotron Radiation Circular Dichroism For RNA Structural Analysis. Methods In Molecular Biology 2113, 135-148.

Lees, J. G., Smith, B. R., Wien, F., Miles, A. J. & Wallace, B. A. (2004). Cdtool-An Integrated Software Package For Circular Dichroism Spectroscopic Data Processing, Analysis, And Archiving. Analytical Biochemistry, 332(2), 285-289.

Malabirade, A., Morgado-Brajones, J., Trepout, S., Wien, F., Marquez, I., Seguin, J., Marco, S., Velez, M. & Arluison, V. (2017). Membrane Association Of The Bacterial Riboregulator Hfq And Functional Perspectives. Sci Rep, 7(1), 10724.

Malabirade, A., Partouche, D., El Hamoui, O., Turbant, F., Geinguenaud, F., Recouvreux, P., Bizien, T., Busi, F., Wien, F. & Arluison, V. (2018). Revised Role For Hfq Bacterial Regulator On DNA Topology. Sci Rep, 8(1), 16792.

Marrington, R., Dafforn, T. R., Halsall, D. J., Macdonald, J. I., Hicks, M. & Rodger, A. (2005). Validation Of New Microvolume Couette Flow Linear Dichroism Cells. The Analyst, 130(12), 1608-1616.

Patterson, T. A. & Dean, M. (1987). Preparation Of High Titer Lambda Phage Lysates. Nucleic Acids Research, 15(15), 6298.

Refregiers, M., Wien, F., Ta, H. P., Premvardhan, L., Bac, S., Jamme, F., Rouam, V., Lagarde, B., Polack, F., Giorgetta, J. L., Ricaud, J. P., Bordessoule, M. & Giuliani, A. (2012). DISCO Synchrotron-Radiation Circular-Dichroism Endstation At SOLEIL. J Synchrotron Radiat, 19(Pt 5), 831-835.

Salivar, W. O., Tzagoloff, H. & Pratt, D. (1964). Some Physical-Chemical And Biological Properties Of The Rod-Shaped Coliphage M13. Virology, 24, 359-371.

Taghbalout, A., Yang, Q. & Arluison, V. (2014). The Escherichia Coli RNA Processing And Degradation Machinery Is Compartmentalized Within An Organized Cellular Network. Biochem J, 458(1), 11-22.

Turbant, F., O., E. H., Partouche, D., Sandt, C., Busi, F., Wien, F. & Arluison, V. (2021). Identification And Characterization Of The Hfq Bacterial Amyloid Region DNA Interactions. BBA Advances, 1, 100029.

Wegrzyn, G., Wegrzyn, A., Konieczny, I., Bielawski, K., Konopa, G., Obuchowski, M., Helinski, D. R. & Taylor, K. (1995). Involvement Of The Host Initiator Function Dnaa In The Replication Of Coliphage Lambda. Genetics, 139(4), 1469-1481.

Wien, F., Martinez, D., Le Brun, E., Jones, N. C., Vronning Hoffmann, S., Waeytens, J., Berbon, M., Habenstein, B. & Arluison, V. (2019). The Bacterial Amyloid-Like Hfq Promotes In Vitro DNA Alignment. Microorganisms, 7(12).

Wien, F., Paternostre, M., Gobeaux, F., Artzner, F. & Refregiers, M. (2013). Calibration And Quality Assurance Procedures At The Far UV Linear And Circular Dichroism Experimental Station DISCO. J. Phys.: Conf. Ser., (425), 122014.

Wien, F. & Wallace, B. A. (2005). Calcium Fluoride Micro Cells For Synchrotron Radiation Circular Dichroism Spectroscopy. Appl Spectrosc, 59(9), 1109-1113.

Yadav, I., Rosencrans, W., Basak, R., Van Kan, J. A. & Van Der Maarel, J. R. C. (2020). Intramolecular dynamics of dsDNA confined to a quasi-one-dimensional nanochanne. Phys. Rev. Research 2, 013294.
