# Supplementary material for: Amyloid-like Hfq interaction with single-stranded DNA: involvement in recombination and replication in Escherichia coli
Source: QRB Discov. 2022 Sep 7;3:e15. doi: 10.1017/qrd.2022.15 (PMC10392684; doi:10.1017/qrd.2022.15)
Supplement: Supplementary file 1 [file qrdsup.zip › S2633289222000151sup002.pdf]

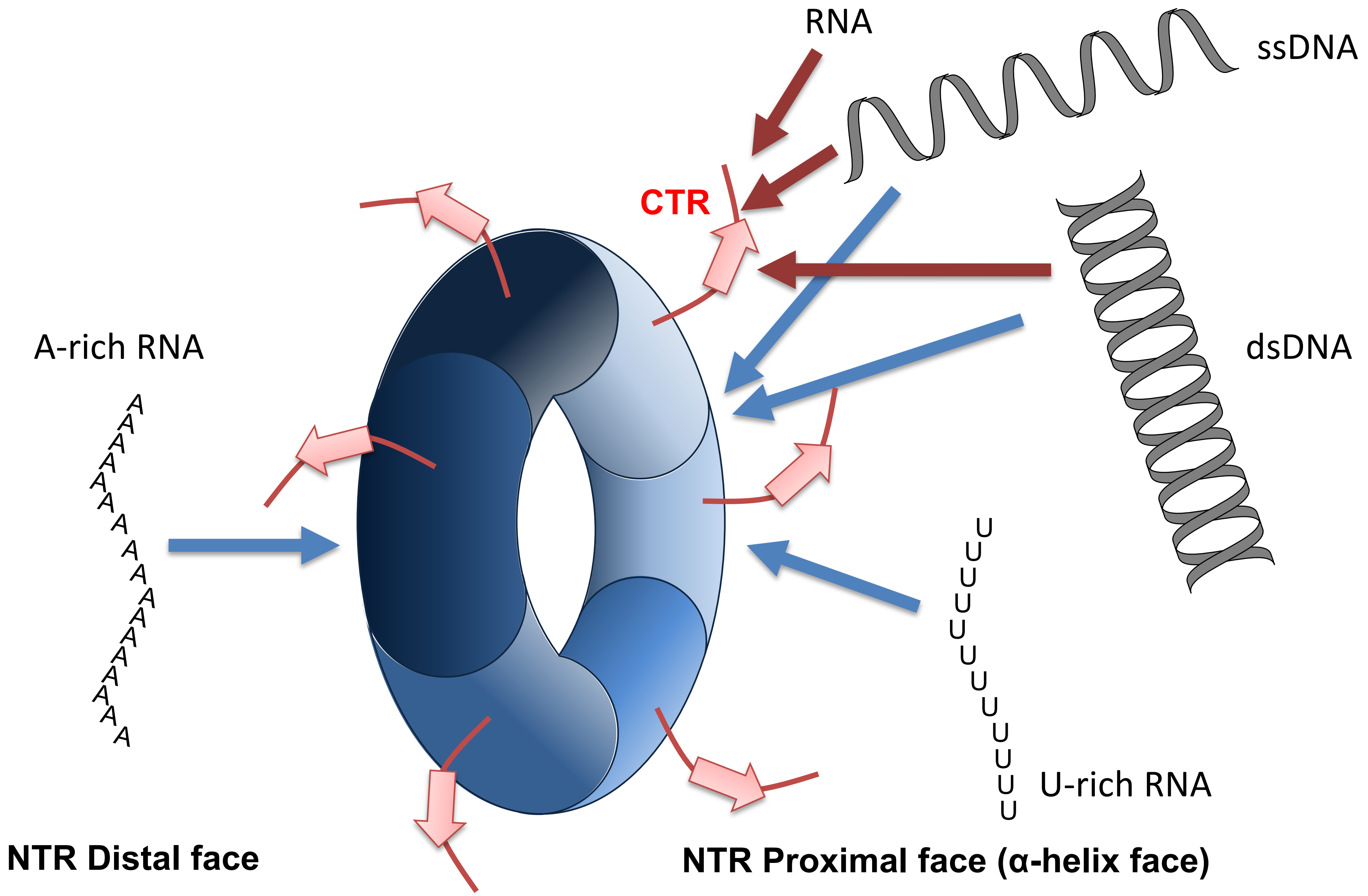

Sup Figure S1: Interaction between Hfq NTR and CTR with nucleic acids. Uridine-rich RNA are bound to the proximal face, while the A-rich sequences bind to the opposite distal face; dsDNA is also bound on the proximal face of the ring. Additionally, CTR binds RNA, ss- and dsDNA.

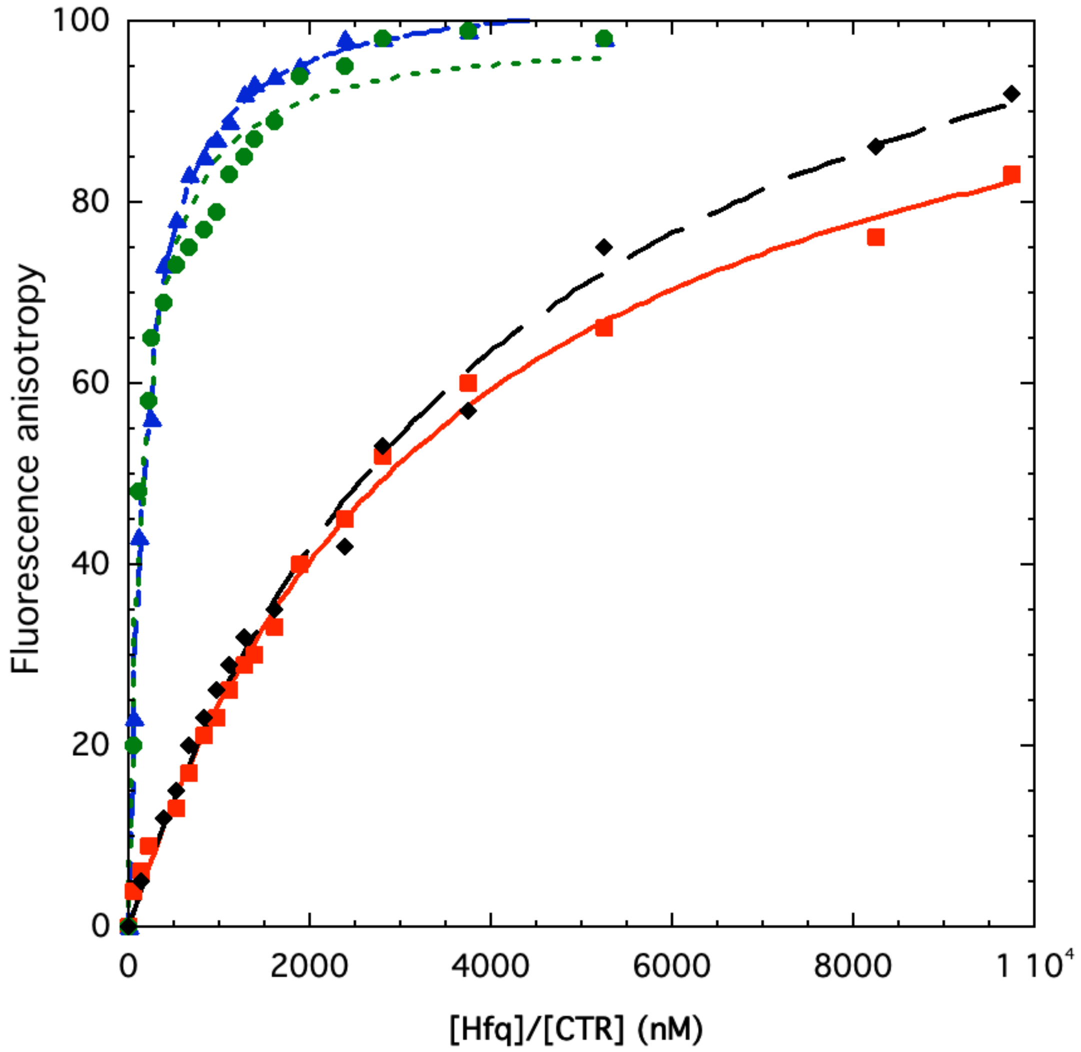

Sup Figure S2: Fluorescence anisotropy measurements using dA_n oligonucleotides. Fluorescence anisotropy measurements were performed using 5'-fluorescein-modified oligonucleotides. Titrations of dA_7 (squares), dA_{20} (triangles) and dA_{59} (circles)with Hfq gave K_d values of 3.5±0.2 µM, 183±8 nM and 166±16 nM, respectively. A weaker affinity is measured for CTR and dA_{59} (rhombuses) and a K_d value of 4.2±0.3 µM.
